# Lambert’s Physiological Series

**Published:** 1851-09

**Authors:** 


*Lambert's Physiological Series.*—We have, for many years, on all suitable occasions, advocated the propriety and importance of popularizing the study of Physiology, by incorporating it among the branches of knowledge taught at Collegiate and Academic Institutions. Attention to the subject by the friends of education has been increasing, and, proportionably, progress has been made in effecting its introduction into our higher schools. It must be sufficiently obvious that, as a province of natural science, it has intrinsic claims, irrespective of its medical relations, certainly not inferior to those conceded to the sister sciences, Chemistry and Physics. In addition to these, as members of the medical profession, the subject is of interest to us, inasmuch as the more well educated, or well informed persons know of the structure and functions of the body, the more they will respect medicine as a science, and the better will they discriminate between the scientific physician, and the ignorant pretender. That this is the legitimate effect we do not doubt. A different opinion, however, is entertained by some. It is thought that to be acquainted with physiology will make persons self-sufficient and assuming, in matters pertaining to pathology and therapeutics. A mere smattering of knowledge is doubtless likely to lead to such a result; but not a thorough acquaintance with the constitution and laws of the human system. We should be glad if in every community there were a host of *lay* physiologists. It would tend to secure a higher grade of education for medical practitioners, for the patient well versed in the healthy phenomena of the economy, would require not less information, in this branch of the science, in his physician, than he himself possessed; and if found deficient in physiological knowledge, it would be a natural and a just inference, that there was a corresponding deficiency in the more practical departments. Let the educated classes of society be competent to gauge the physiological acquirements of practitioners, and something would be accomplished toward consigning to a proper relative position, those who pass their leisure hours in bar-rooms and stables, and yet claim to be posted up, equally with the most industrious, in all that relates to a science so progressive as medicine!

These remarks have been suggested by a series of works on Physiology, designed for schools, by T. S. Lambert, M. D. The series consists of a *first*, a *second*, and a *third* book, prepared for classes in successive stages of advancement. It is highly important that works of this description should be free from errors, and embody the latest discoveries and improvements in the science. Several works for schools, and the popular reader, have been issued within a few years. One of the best of these is a treatise by Prof. C. A. Lee, which has, deservedly, an extensive circulation.

We regret to state that all the works of a popular character which have been employed to a greater or less extent as text books, are not unobjectionable on the score of accuracy. This is the case with a work, noticed in a former volume of this journal, by Dr. Cutter. Our attention has been called to several gross errors in that work.

So far as we have examined the series by Dr. Lambert, they appear free from objections as respects correctness, and they seem well adapted to the objects for which they are designed.

Dr. Lambert is engaged in editing a complete American edition of Muller's Physiology, which is to be issued in numbers, and furnished at the actual cost of publication.

The same industrious author has superintended a republication of anatomical plates for illustrating physiological subjects, which are elegant, and may be purchased at a very cheap rate.

